# ECG J waves

**DOI:** 10.4103/0974-2700.43200

**Published:** 2008

**Authors:** Matt Sisko, Bradley F Peckler

**Affiliations:** 1 College of Medicine, University of South Florida, Bruce B Downs Blvd, Tampa - 33601, FL – USA; 2 Department of Medicine, Division of Emergency Medicine, University of South Florida, 2 Columbia Dr, # 504, Tampa - 33606, FL – USA

**Figure 1:**
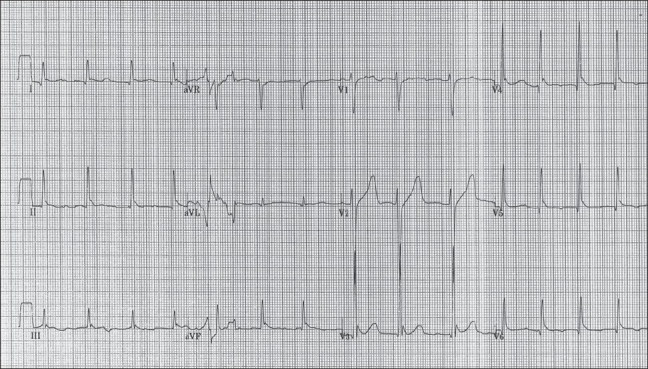
The ECG obtained on presentation

A 19-year-old snow skier was found unconscious by a rescue team after a 2-day search. He was bradycardic, dehydrated, and had sluggish dilated pupils bilaterally. There was no evidence of trauma. The patient was found to have a core body temperature of 82°F (27.7°C). An electrocardiogram (ECG) was obtained [Figure 1] and this showed the classic ECG changes seen in hypothermia.

Osborne waves, also referred to as J waves, were first described by Tomaszewkski in 1938. The J wave is a positive convex deflection that occurs at the junction of the QRS complex and ST segment, the J-point. They occur most prominently in the inferior leads: II, III, and aVF and the precordial leads: V_5_ – V_6_ when the core body temperature falls below 32°C (89.6°F). The magnitude of the deflection above the isoelectric line varies inversely with the fall in core body temperature below 32°C. Computer interpretation of ECG has been found to be unreliable in hypothermia, with the J waves in some cases being mistaken for myocardial injury current. When J waves are seen the differential diagnosis includes hypercalcemia, sepsis, CNS lesion, cardiac ischemia, and Brugada syndrome.

The pathophysiology of the J wave is not well understood but it is theorized that the hypothermic state causes an increased repolarization response in phase 1 of the epicardial action potential due to effects on voltage-gated potassium channels. J waves are relatively specific, being seen in 80% of hypothermic patients and are therefore diagnostic. They are not, however, considered pathognomonic as they have also been reported in normothermic patients. In addition to J waves, other nonspecific ECG findings seen in hypothermia include atrial fibrillation and QT interval prolongation. Treatment of the underlying hypothermia by rewarming will cause the J waves to resolve when the core body temperature rises above 32°C. Treatment modalities include passive and active external rewarming for mild to moderate hypothermia, with core rewarming being reserved for severe hypothermia.
